# A unified STR profiling system across multiple species with whole genome sequencing data

**DOI:** 10.1186/s12859-019-3246-y

**Published:** 2019-12-20

**Authors:** Yilin Liu, Jiao Xu, Miaoxia Chen, Changfa Wang, Shuaicheng Li

**Affiliations:** 1 Liaocheng Research Institute of Donkey High-Efficiency Breeding and Ecological Feeding, Liaocheng University, Liaocheng, China; 2 City University of Hong Kong, 83 Tat Chee Ave, Kowloon Tong, Hong Kong, China

**Keywords:** Short tandem repeats, Whole genome sequencing, Individual identification

## Abstract

**Background:**

Short tandem repeats (STRs) serve as genetic markers in forensic scenes due to their high polymorphism in eukaryotic genomes. A variety of STRs profiling systems have been developed for species including human, dog, cat, cattle, *etc*. Maintaining these systems simultaneously can be costly. These mammals share many high similar regions along their genomes. With the availability of the massive amount of the whole genomics data of these species, it is possible to develop a unified STR profiling system. In this study, our objective is to propose and develop a unified set of STR loci that could be simultaneously applied to multiple species.

**Result:**

To find a unified STR set, we collected the whole genome sequence data of the concerned species and mapped them to the human genome reference. Then we extracted the STR loci across the species. From these loci, we proposed an algorithm which selected a subset of loci by incorporating the optimized combined power of discrimination. Our results show that the unified set of loci have high combined power of discrimination, >1−10^−9^, for both individual species and the mixed population, as well as the random-match probability, <10^−7^ for all the involved species, indicating that the identified set of STR loci could be applied to multiple species.

**Conclusions:**

We identified a set of STR loci which shared by multiple species. It implies that a unified STR profiling system is possible for these species under the forensic scenes. The system can be applied to the individual identification or paternal test of each of the ten common species which are *Sus scrofa* (pig), *Bos taurus* (cattle), *Capra hircus* (goat), *Equus caballus* (horse), *Canis lupus familiaris* (dog), *Felis catus* (cat), *Ovis aries* (sheep), *Oryctolagus cuniculus* (rabbit), and *Bos grunniens* (yak), and *Homo sapiens* (human). Our loci selection algorithm employed a greedy approach. The algorithm can generate the loci under different forensic parameters and for a specific combination of species.

## Background

Short tandem repeats (STRs), also referred to as simple sequence repeats or microsatellite loci, are DNA fragments made up of tandem repeats of 1–6 bp sequence units [1, 2]. STRs are ubiquitous in eukaryotic genomes and are highly polymorphic. They are widely adopted as genetic markers in human [3] and non-human forensic applications such as individual identification [4, 5], paternity testing [6, 7], and kinship analysis [8]. DNA profiles can provide valuable clues and evidence during investigations of crime scenes. In previous studies, a number of STR profiling systems have been developed for various animal species including dog [9, 10], cat [11, 12], horse [13, 14], *etc*. However, to maintain one DNA profiling system for each species can be tedious and costly. In previous studies [15–17], panels of cross-species STR markers were developed among some closely related species. Given the fact that mammalian animals share large proportions of DNA sequences [18, 19], it is sensible to expect that a unified set of DNA markers may exist and the set can be identified from the shared sequence regions, and hence, a unified STR profiling system is possible for multiple species.

In this study, we aim to develop a unified set of STR loci for ten species, namely *Sus scrofa* (pig), *Bos taurus* (cattle), *Capra hircus* (goat), *Equus caballus* (horse), *Canis lupus familiaris* (dog), *Felis catus* (cat), *Ovis aries* (sheep), *Oryctolagus cuniculus* (rabbit), and *Bos grunniens* (yak), and *Homo sapiens* (human). Our objective is to identify a minimum set of STR loci satisfying the forensic parameters from the shared genome regions among these species. Previous studies have utlized high-volume data from the shotgun sequencing technology in forensic analysis to search the new forensic DNA markers [20, 21]. While our targeted species belong to different families or different orders, and it is challenging and time-consuming to obtain the their shotgun data. Therefore, we proposed a model based on whole genome sequence data.

In this work, we downloaded the whole genome sequence (WGS) data of the multiple species. Then, we mapped the sequencing data with BWA [22] to the human genome (hg19) [23]. After that, we obtained the possible STR sites in each species using the software package *lobSTR* [24]. Last, we performed a greedy locus selection algorithm with the incorporation of the forensic parameters to identify a unified set.

The obtained locus set contains much less loci than the total number of the loci from individual species. The acquired loci set also demonstrates a lower random-match probability and a higher combined power of discrimination when applied to each species. Our statistical results show that the ultimately unified loci set has the *combined power of discrimination* (CPD, $\mathbb {C}(\mathbb {L}$)) larger than 1−10^−9^ for each involved species, and random-match probability at most 6.30×10^−8^. Furthermore, our proposed loci selection algorithm can also be applied to individual species. The experimental results showed that, in comparison with other previous report work, the proposed method is capable to generate more efficient loci for the given species. Using the $\mathbb {C}'(\mathbb {L})$ and $\mathbb {R}(\mathbb {L})$ values in 13 CODIS loci as benchmarks, the proposed algorithm generated eight loci to meet the criteria with lower $\mathbb {C}'(\mathbb {L})$ and $\mathbb {R}(\mathbb {L})$, indicating that the selected loci are more sensitive for individual identification.

## Methods

### Datasets

We downloaded next-generation sequencing (NGS) data of the ten species from NCBI (*Sus scrofa*: SRP028348; *Bos taurus*: SRP039339; *Capra hircus*: SRP102144, SRP108014; *Equus caballus*: SRP061963, SRP067684, ERP010944, ERP011292; *Canis lupus familiaris*: SRP035294; *Felis catus*: SRP061392, SRP093-936; *Ovis aries*: SRP013208; *Oryctolagus cuniculus*: SRP053211, ERP111038; *Bos grunniens*: SRP059061), and data from 1000 Genome project [25] for human being. The involved species belong to different families or even different orders.

In order to obtain the candidate STR loci in the shared genomic region of involved species, we aligned WGS reads in the samples to hg19 and processed the aligned data with *lobSTR*. According to the outputs of *lobSTR*, we estimated allele frequencies as well as genotype frequencies of sites in each species, which were used to estimate forensic parameters described in the following sections.

### Problem statement

In this work, we call the multiple species, which we want to search the unified STR locus set from, as an *integrated pseudo species*. Given the genome data of multiple species, the main purpose here is to obtain a small set of STR loci from the shared genome region among the species to achieve individual identification; that is, we want to find a set of STR loci with the cardinality minimized. In addition, these loci should satisfy the criteria specified by the forensic parameters for both an individual species and the integrated pseudo species.

#### Call rate

In each species, the *call rate* at each locus (denoted as *η*_*ℓ*_) is the percentage of the individuals covers the locus in the sample data. The call rate is restricted by the pre-specified threshold *δ*_*η*_ so that the selected loci $\mathbb {L}_{k}$ could be observed in the majority of the individuals in a species.

#### Parameters for high distinguishing power

First, within each species, a valid locus should satisfy the constraints set by four forensic parameters: the *power of discrimination* (PD), the *probability of matching* (PM), the *power of exclusion* (PE), and *heterozygosity* (HE) [26]. The constraints set by the parameters to ensure that only loci with high distinguishing power on individuals would be considered. Denote PD, PM, PE, and HE of the locus *ℓ* of the species *s* as *D*_*ℓ*,*s*_, *M*_*ℓ*,*s*_, *E*_*ℓ*,*s*_, *H*_*ℓ*,*s*_, respectively. Let thresholds of the four parameters as *δ*_*d*_, *δ*_*m*_, *δ*_*e*_, and *δ*_*h*_, the selected loci set $\mathbb {L}_{s}$ of each species *s* should satisfy

1 $$\qquad \left\{\begin{array}{ll} {D_{\ell, s}} \ge \delta_{d} \\ {M_{\ell, s}} \le \delta_{m} \\ {E_{\ell, s}} \ge \delta_{e} \\ {H_{\ell, s}} \ge \delta_{h} \end{array}\right.,\quad\ell \in \mathbb{L}_{s}   $$

Moreover, since multiple species are considered here, we need to define the PD for the integrated pseudo species at each locus. At locus *ℓ*, denote PD for the integrated pseudo species as $\mathbb {D}_{\ell }$, then the calculation of $\mathbb {D}_{\ell }$ is defined as

2 $$ \qquad \mathbb{D}_{\ell} = 1 - \prod_{s}{M_{\ell,s}} = 1 - \prod_{s} {(1 - D_{\ell, s})}.   $$

#### Combined power of discrimination ($\mathbb {C}(\mathbb {L}$))

$\mathbb {C}(\mathbb {L}$) is also a common parameter to evaluate the capability of individual identification of a locus set. Denote $\mathbb {C}(\mathbb {L}$) for species *s* as *C*_*s*_. Then,

3 $$ C_{s}(\mathbb{L}) = 1 - \prod_{\ell \in \mathbb{L}} {M_{\ell,s}} = 1 - \prod_{\ell \in \mathbb{L}}({1 - D_{\ell,s}}).   $$

The cumulative product of PM refers to the *combined probability of matching* (CPM) and maximizing the value of $\mathbb {C}(\mathbb {L}$) is equivalent to minimizing the value of CPM($1 - \mathbb {C}(\mathbb {L})$, denote it as $\mathbb {C}'(\mathbb {L})$).

Also, we also define the CPD for the integrated pseudo species. Similar to the definition above, for the integrated population, $\mathbb {C}(\mathbb {L}$) is defined as

4 $$ \qquad \mathbb{C}(\mathbb{L}) = 1 - \prod_{\ell \in \mathbb{L}}{(1 - \mathbb{D}_{\ell})}   $$

We aim to find a locus set $\mathbb {L}$ to satisfy $C_{s}(\mathbb {L})\ge \delta _{c}$ for each individual species as well as optimize the value of $\mathbb {C}$ for the integrated pseudo species.

#### Random-match probability (RMP)

*Random-match probability* (RMP, $\mathbb {R}(\mathbb {L})$), defined as the probability that a randomly selected individual from the population other than the suspect would have the given DNA profile, is another criterion to evaluate DNA profile systems. Such constraints are imposed to statistically eliminate the fallacy that an irrelevant individual in the population is matched to the given profile [26]. Therefore, here we limit value of $\mathbb {R}(\mathbb {L})$ to be under 1/*N* where *N* refers to the number of individuals in the population on which we would like to apply our selected markers. Denote the *maximum profile frequency* (MPF) at each locus *ℓ* of species *s* as *F*_*ℓ*,*s*_. Then the $R_{s}(\mathbb {L})$ of locus set $\mathbb {L}$ in species *s* is calculated as

5 $$ \qquad\quad R_{s}(\mathbb{L})=\prod_{\ell \in \mathbb{L}} {F_{\ell, s}}   $$

We want to find a locus set $\mathbb {L}$ such that for each species *s*, $R_{s}(\mathbb {L})\le \frac {1}{N_{s}}$; that is, the cumulative product of is set to be no more than the reciprocal of the population size. Similarly, we can define the the maximum profile frequency and the random-match probability for the integrated pseudo species, and denote them as $\mathbb {F}_{\ell }$ and $\mathbb {R}(\mathbb {L})$, respectively.

The loci apply to both individual or the integrated pseudo species; it is necessary to restrict $\mathbb {R}(\mathbb {L})$s for both in individual species and in the integrated pseudo species below the threshold *δ*_*r*_, the reciprocals of the population sizes, for both a single species or the integrated pseudo species.

### Locus selection

First, we aligned raw sequencing data to the reference of the human genome, processed the data through *lobSTR* workflow and obtained STR locus candidate set from in the overlapping genomic area of the involved species. We excluded loci on the sex chromosome, and mononucleotide repeats, which are inapplicable in practical situations. Using loci retrieved by *lobSTR*, we estimated allele frequency at each locus and used the frequencies to compute PD, PM, PE, HE, as well as maximum genotype frequency. Then we applied the thresholds specified in Eq. (1) on PD, PM, PE, HE and call rate to filter the STR loci.

The selected loci set should have the $C_{s}(\mathbb {L}$) values no less than *δ*_*c*_ for each species, and its $R_{s}(\mathbb {L})$s are no more than *δ*_*r*_ for each species and an optimized $\mathbb {C}(\mathbb {L}$) for the integrated pseudo species. The $\mathbb {R}(\mathbb {L})$ can be expressed as the products of $\mathbb {F}_{\ell }$ values, and $\mathbb {C}'(\mathbb {L})$ can be expressed as the products of PM values.

Next, we employ a greedy algorithm to find a unified STR locus set from the filtered loci. We started from an empty set of loci. At each iteration, we incorporate the locus which reduces $1-\mathbb {C}$ (denote it as $\mathbb {C}'$) and $\mathbb {R}$ the most into the set. As both $\mathbb {C}'$ and $\mathbb {R}$ are probabilities, we choose the locus which decreases their product the most. We repeat the process until the $\mathbb {C}'$ and $\mathbb {R}$ are below the pre-specified thresholds. The algorithm is displayed as (Algorithm 1). To initialize, we create two vectors *v*_*C*_ and *v*_*R*_, with initial values as 1. They will store the values of *M*_*ℓ*,*s*_) and *F*_*ℓ*,*s*_ of all loci in every species. *v*_*C*_(*s*) and *v*_*R*_(*s*) are the *M*_*ℓ*,*s*_ and *F*_*ℓ*,*s*_ values of species *s* of the current identified loci. Once a species *s* has its $C_{s}'(\mathbb {L})$ (or $R_{s}(\mathbb {L})$) reach the given thresholds, we skip the species for later iterations.


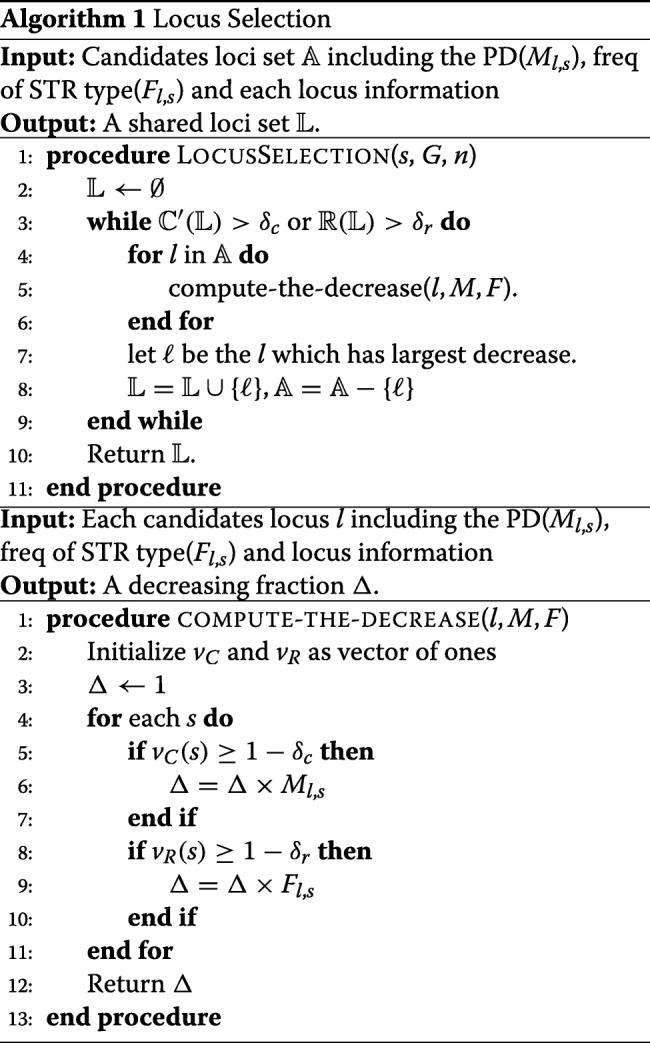


### Sample size determination

According to Hale et. al [27], most of the informative alleles, alleles at a frequency of ≥0.05, could be captured by 30 samples, thus the estimated allele frequencies from 30 samples can represent the population allele frequencies. Here, we utilized the data of the Phase 3 publication of the 1000 Genomes Project, and randomly sampled different numbers of individuals from the data set. The statistical results showed that with 25 or more samples, at least 90% of alleles at each site can be detected, and that the difference between the estimated and theoretical values of PD, HE and MPF is small–less than 0.1.

## Results

### Performance on pseudo species consisting of 10 species

**Fig. 1:**
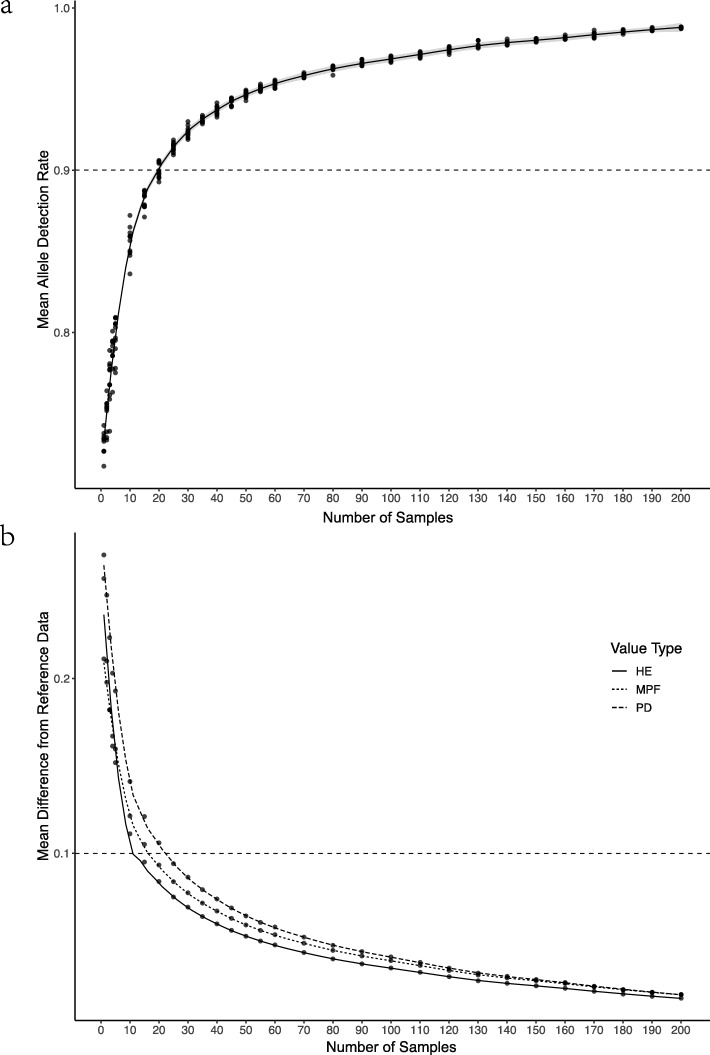
Impact of sample size on allele detection and major forensic parameters (PD, HE, MPF)

Prior to the locus selection process, we conducted experiments to examine impact of different sample sizes on the detection of alleles as well as estimation of the involved forensic parameters. We chose the data in the 1000 Genomes Project. We sampled different numbers of individuals from this population and estimated number of alleles detected at each site, as well as the forensic parameters (PD, HE, MPF) involved in individual identification. As shown in Fig. 1, with 25 or more samples, over 90% of alleles can be detected and meanwhile, the difference between the estimated and theoretical values of forensic parameters are small. Therefore, using 25–30 samples will also be able to generate sensible estimations.

**Fig. 2:**
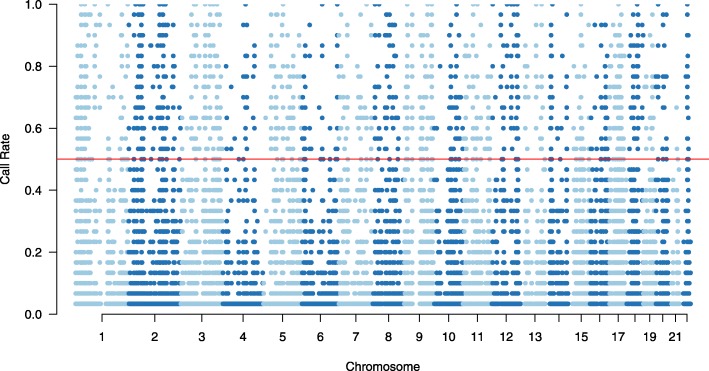
The distribution of call rate

After processing data through *lobSTR*, 119,717 loci were detected with reference to human genome of all 23 chromosome pairs. The initial distribution of call rate among STR loci is shown in Fig. 2.

**Fig. 3:**
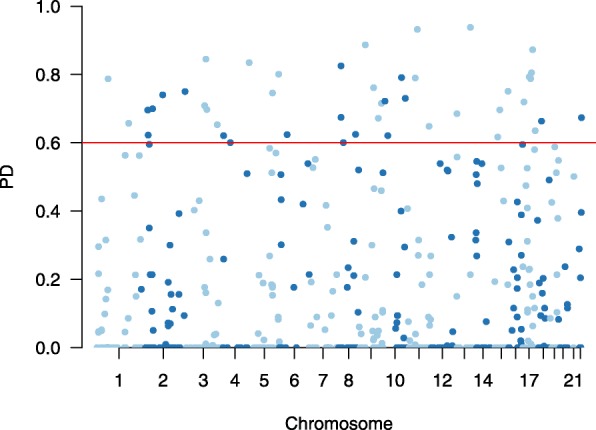
Distribution of PD of remaining loci (*ηl*≥0.5)

First, we filtered out loci that were observed only in single species, 7784 STR loci remains. Next, we excluded mononucleotide repeat loci as well as loci on sex chromosomes, and 4614 loci remain. After we dropped loci with call rate less than the threshold *δ*_*η*_ and filtered out loci that failed to satisfy any of the thresholds of forensic parameters (*δ*_*d*_=0.6,*δ*_*m*_=0.4,*δ*_*e*_=0.3,*δ*_*h*_=0.5), we obtained 1268 loci subjected to further processing. The distribution of PD and genotype frequency among remaining loci is shown in Figs. 2 and 3. To be noted, in Fig. 3 the y-axis refers to the joint PD defined in (2), while in Fig. 2, the y-axis represents a subtraction of the cumulative value of genotype frequency at each locus. The red lines Figs. 2 and 3 stand for our desired thresholds for these two parameters in the loci selection algorithm.

We applied the selection algorithm on STR sites of the concerned species. We set the threshold for $\mathbb {C}(\mathbb {L}$) at 0.9999 and $\mathbb {R}(\mathbb {L})$ at 10^−7^ for every individual species and generated a set with 31 loci for ten species. Table 1 shows forensic parameters of selected loci with highest PDs, and Table 2 shows assessment of selected loci on their combined power for individual identification as well as paternity testing on each species population. The generated loci set has $\mathbb {C}(\mathbb {L}$)s higher than 1−10^−9^, $\mathbb {R}(\mathbb {L})$s less than 10^−7^ for every concerned species, suggesting its power for distinguishing individuals randomly chosen from a related species. Also, to consider the possible use of generated loci set in paternity testing, we evaluated the combined power of exclusion (CPE) within each species population. We can observe that CPEs are higher than 0.99 for every species group.

**Table 1:** Forensic parameters of selected loci

CHR	POS	RUL	HE	PD
chr17	7787390	2	0.814716312056738	0.935450047622492
chr14	57279935	2	0.827094474153298	0.932795344883323
chr11	57467882	2	0.828629032258065	0.932229995727539
chr1	207997377	2	0.785381285381285	0.923859365077692
chr11	46142707	2	0.8	0.917120154272911
chr16	4322949	2	0.782472613458529	0.913962030606996
chr15	98292738	2	0.777777777777778	0.901665702815812
chr3	119541091	2	0.780241935483871	0.900781631469727
chr15	73889791	5	0.767195767195767	0.891822417742607
chr2	39201053	2	0.747619047619048	0.881054955418381
chr2	241696826	3	0.747619047619048	0.875768032693187
chr12	118588317	5	0.758241758241758	0.873070486988594
chr9	14086350	4	0.734042553191489	0.86962890625
chr6	163992752	6	0.719774011299435	0.868980555555556
chr5	124081853	2	0.733333333333333	0.863633976401387
chr8	22619315	2	0.701149425287356	0.856881481481481
chr18	72357594	2	0.683257918552036	0.856743570778334
chr2	135703320	3	0.711693548387097	0.851266860961914
chr2	36777760	6	0.68974358974359	0.8426265625
chr17	49255307	3	0.727272727272727	0.842592592592593
chr3	114173776	2	0.727272727272727	0.842592592592593
chr3	137413014	2	0.666666666666667	0.821603869787472
chr16	22092942	2	0.692307692307692	0.814504373177843
chr3	114033630	3	0.674645390070922	0.810491491247107
chr4	54876122	6	0.634765294711289	0.808673104516968
chr1	176522684	2	0.712121212121212	0.805266203703704
chr22	36140123	4	0.67032967032967	0.786182840483132
chr1	27108339	5	0.62145390070922	0.77700524691358
chr3	160219801	2	0.591666666666667	0.757476806640625
chr4	17885278	2	0.546654861535651	0.68947775749674
chr3	187439732	2	0.533333333333333	0.6144

**Table 2:** Evaluation of selected loci on each species

Species	$\mathbb {R}(\mathbb {L})$	$\mathbb {C}(\mathbb {L}$)	CPE
cat	5.0615×10^−10^	0.99999999999993383	0.99978049249880108
cattle	1.0536×10^−8^	0.99999999998633915	0.99794653711292580
dog	3.9239×10^−10^	0.99999999999975098	0.99970843603702098
goat	2.3005×10^−8^	0.99999999997592071	0.99857509526114097
horse	5.0166×10^−8^	0.99999999977198839	0.99625817962597518
human	3.3695×10^−8^	0.99999999999140876	0.99933064560113372
pig	6.2908×10^−8^	0.99999999985080279	0.99713785294534030
rabbit	4.4219×10^−10^	0.99999999999992795	0.99980453489094234
sheep	5.0696×10^−10^	0.99999999999990974	0.99975253609811832
yak	1.9169×10^−9^	0.99999999999823685	0.99931237001520157

**Fig. 4:**
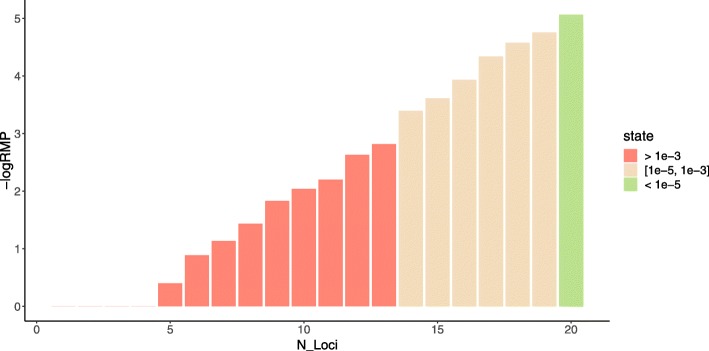
$\mathbb {R}(\mathbb {L})$ achieved by different number of loci

**Fig. 5:**
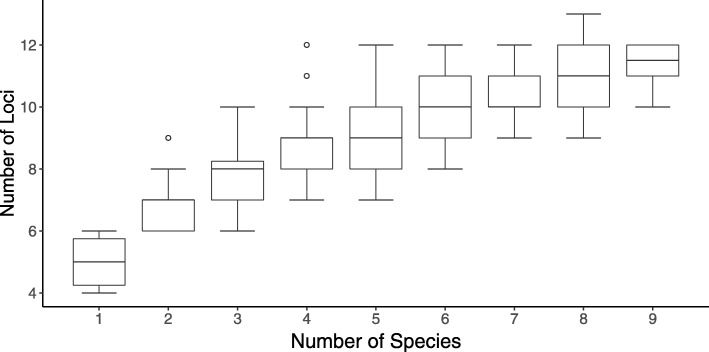
Number of loci generated for different number of species

In addition, our algorithm can be applied with different thresholds and on a different number of species. We applied the selection algorithm on 10 species with $\mathbb {C}(\mathbb {L}$) threshold ranging from 1−10^−3^ to 1−10^−7^ and $\mathbb {R}(\mathbb {L})$ threshold from 10^−3^ to 10^−10^. As shown in Table 3, at a given $\mathbb {C}(\mathbb {L}$) threshold, the number of loci would increase with descending threshold of $\mathbb {R}(\mathbb {L})$; conversely, when the threshold of $\mathbb {R}(\mathbb {L})$ is set, the number of loci may slightly increase or remain unchanged when more rigorous $\mathbb {C}(\mathbb {L})$ threshold is given. Moreover, we examined the value of $\mathbb {R}(\mathbb {L})$ that different number of loci could achieve among ten species. (Figure 4) It can be seen that to satisfy $\mathbb {R}(\mathbb {L})$ upper bounded at 10^−5^ in each species, at least 21 loci are required. At given $\mathbb {C}(\mathbb {L}$) and $\mathbb {R}(\mathbb {L})$ threshold, the number of loci generated among different number of species is shown in Fig. 5. It can be seen that when $\mathbb {R}(\mathbb {L})$ threshold is settled, the increment of species number will not result in continuously growing of loci number, indicating that our proposed algorithm is effective when more species groups are involved.

**Table 3:** Number of loci selected under different thresholds of $\mathbb {R}(\mathbb {L})$ and $\mathbb {C}(\mathbb {L}$)

	1−10^−3^	1−10^−4^	1−10^−5^	1−10^−6^	1−10^−7^
10^−3^	12	12	14	17	17
10^−4^	16	16	16	17	17
10^−5^	21	20	20	21	21
10^−6^	27	27	27	25	25
10^−7^	31	31	31	31	31
10^−8^	36	36	36	36	36
10^−9^	41	41	41	41	41
10^−10^	46	46	46	46	46

### Our method beats CODIS on human

**Fig. 6:**
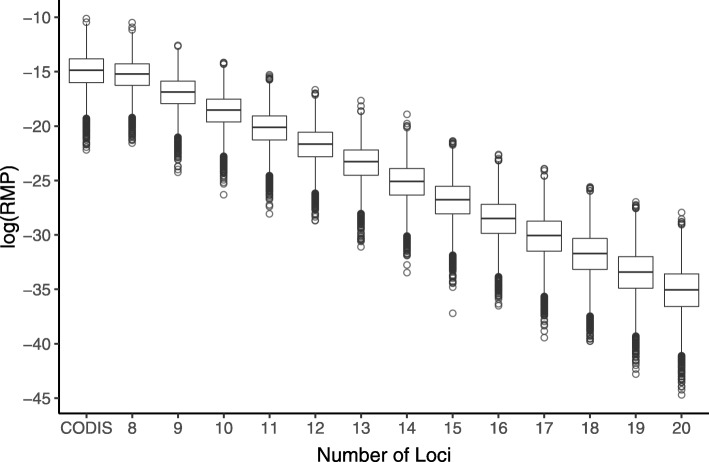
Box plot for common logarithms of RMPs on 10,000 simulated individuals with CODIS and loci selected with proposed method

The Federal Bureau of Investigation (FBI) has published thirteen core loci for the Combined DNA Index System (CODIS)[28] in 1997, which have been used as dominant DNA markers in human profiling. Here, we retrieved the thirteen loci from 320 individuals of Chinese population in 1000 Genome Project. We computed the $\mathbb {C}(\mathbb {L})$ and $\mathbb {R}(\mathbb {L})$ of CODIS based on the data. To evaluate the performance of the proposed algorithm, we used the calculated values of $\mathbb {C}(\mathbb {L})$ and $\mathbb {R}(\mathbb {L})$ as thresholds, and applied the algorithm on the same population. Eight loci were selected by our algorithm to satisfy both thresholds. The fewer number of loci suggests that our algorithm is effective in searching for loci to meet given thresholds. As shown in Table 4, the selected loci has higher $\mathbb {C}(\mathbb {L})$ and lower $\mathbb {R}(\mathbb {L})$ than the CODIS loci. To further evaluate the efficacy of the proposed algorithm in loci selection, we generated sets containing different number of loci for the same Chinese population. For each generated loci set $\mathbb {L}$, we simulated profiles of 10,000 individuals at each selected locus, and computed the values of $\mathbb {R}(\mathbb {L})$. The results are shown in Fig. 6. With more loci chosen by the proposed algorithm, the value of $\mathbb {R}(\mathbb {L})$ would decrease accordingly. When a locus set with 13 loci was generated, the $\mathbb {R}(\mathbb {L})$ became much smaller than CODIS, suggesting the much lower probability of occurrence of random matching cases. Therefore, it can be concluded that the greedy strategy implemented in the proposed algorithm - to assign priority on loci with high PD and low MPF - is capable to generate a small locus set to achieve pre-specified CPD and RMP on target population.

**Table 4:** Forensic parameters of selected loci and loci in CODIS

	Our	CODIS
*N*_*loci*_	8	13
$\mathbb {R}(\mathbb {L})$	4.51×10^−11^	8.42×10^−10^
$\mathbb {C}'(\mathbb {L})$	1.35×10^−14^	4.58×10^−13^

**Fig. 7:**
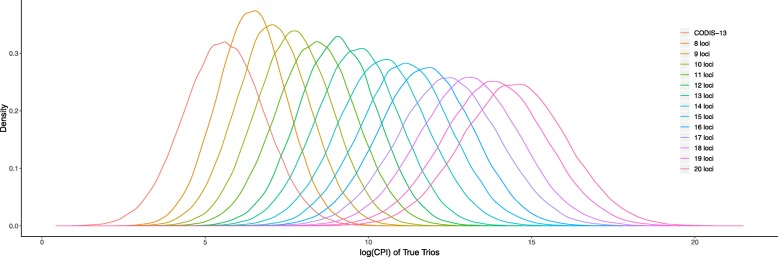
Normalized probability distributions of common logarithm of CPI in trio paternity testing in human

To better evaluate the capability of using the selected loci in paternity test, we conducted simulations of trio paternity testing cases and for human population. We generated sets containing different number of loci - ranging from 8 to 20, where the 8-loci set was generated when the observed values of $\mathbb {C}(\mathbb {L})$ and $\mathbb {R}(\mathbb {L})$ of CODIS were used as the thresholds in our selection algorithm. We computed the combined paternity index (CPI) [29] as the evaluation metric. We simulated 1000 true trio families (where the man and woman are true biological parents of the child) as well as 1000 false trio families (where the man is a random male chosen from the population) and calculated the paternity index (PI) and CPI for families. As shown in Fig. 7, we took the logarithm of CPI values. In true trio cases, where CPIs are usually higher than 1, larger CPI indicates the corresponding loci set can provide higher paternity probabilities, and thus more reliable in practical use; while in false trio cases, where CPIs become usually lower than 1, smaller value implies the less probability of false exclusion. It can be seen that, in comparison with CODIS, the 8 loci obtained by our algorithm at same thresholds have higher CPI at true trio case and lower CPI at false trio case. Moreover, when increasing the number of loci selected by our algorithm, the normalized curves go further from the y-axis, suggesting the growing reliability of the selected loci in paternity testing. To sum up, in both terms of individual identification and paternity testing, our proposed algorithm is capable to select efficient loci to generate an optimized loci set for a given population.

### Our method beats Kemp’s on pseudo species consisting of cattle, goat, and sheep

In the work of Kemp et al. [16], a panel of 97 microsatellite markers was developed jointly for cattle, goat, and sheep. Here we extracted samples of cattle, goat, and sheep to form a pseudo species, and applied the selection algorithm on this newly integrated population. With the threshold for $\mathbb {C}(\mathbb {L}$) and $\mathbb {R}(\mathbb {L})$ at 0.9999 and 10^−7^ respectively, 18 loci were selected for the three species. As shown in Table 5, the generated loci set $\mathbb {L}$ has low $\mathbb {R}(\mathbb {L})$ (under 10^−7^), high $\mathbb {C}(\mathbb {L})$ (greater than 1−10^−9^) and CPE (greater than 0.996), indicating the power of selected loci in identity and paternity testing.

**Table 5:** Forensic parameters of loci selected for cattle, goat, and sheep

Species	$\mathbb {R}(\mathbb {L})$	$\mathbb {C}(\mathbb {L}$)	CPE
cattle	3.8319×10^−8^	0.999999999815	0.996642777607
goat	3.4890×10^−8^	0.999999999908	0.998089765874
sheep	4.6930×10^−10^	0.999999999995	0.999261605878

## Conclusion

In this study, we developed an algorithm based on whole genome sequencing (WGS) data to generate a unified STR loci set for multiple species. The algorithm was designed to search for minimum number of loci to optimize the combined power of discrimination ($\mathbb {C}(\mathbb {L}$)) for each species as well as the integrated population. For each individual species, the selected loci should have $\mathbb {C}(\mathbb {L}$) no less than *δ*_*c*_ (here we set *δ*_*c*_=0.9999) to ensure their efficacy to distinguish one individual from another, and random-match probability ($\mathbb {R}(\mathbb {L})$) no greater than *δ*_*r*_ to control the fallacy that two randomly selected individuals would have same profiles at given loci.

We included 10 species in this study, namely, *Sus scrofa* (pig), *Bos taurus* (cattle), *Capra hircus* (goat), *Equus caballus* (horse), *Canis lupus familiaris* (dog), *Felis catus* (cat), *Ovis aries* (sheep), *Oryctolagus cuniculus* (rabbit), *Bos grunniens* (yak), and *Homo sapiens*. To obtained all possible STR sites at the common genome region among involved species, we mapped raw sequencing data of individual samples concerning the human genome. We implemented the proposed selection algorithm on STR sites owned by at least two species and finally generated 31 loci at $\mathbb {R}(\mathbb {L})$ (≤10^−7^) for all concerned species. Under this threshold, the generated loci set has $\mathbb {C}(\mathbb {L}$)s greater than 1−10^−9^ and $\mathbb {R}(\mathbb {L})$s no greater than 10^−7^, which collectively demonstrate its capability of individual identification in every involved species population. Furthermore, we assessed the capacity of using selected loci in paternity testing by their combined power of exclusion (CPE). The generated loci set could achieve CPEs greater than 0.99.

In addition, we evaluated our proposed algorithm by applying it on different selection thresholds and varying number of species. It turns out that the loci number may increase to satisfy more rigorous $\mathbb {R}(\mathbb {L})$ threshold, whereas with settled $\mathbb {R}(\mathbb {L})$ threshold, the number of loci would not continue to increase significantly when more species are involved. Thus it can be concluded that the algorithm proposed here can find loci that commonly have high power of discrimination in involved species and generate a loci set to satisfy the criteria of $\mathbb {C}(\mathbb {L}$) and $\mathbb {R}(\mathbb {L})$ with a minimized number of loci.

With the data from 1000 Genomes Project, we computed the $\mathbb {C}(\mathbb {L}$) and $\mathbb {R}(\mathbb {L})$ of 13 CODIS loci, used the values as thresholds in our proposed loci selection algorithm, and obtained eight loci that could satisfy the criteria. This loci set generated by our method not only have fewer number of loci, but also demonstrate higher $\mathbb {C}(\mathbb {L}$) and lower $\mathbb {R}(\mathbb {L})$ in the concerned population. In addition, in the respective simulated 1000 cases of true trio and false trio paternity tests, the generated 8-loci set demonstrated higher reliability than CODIS in terms of the combined paternity index (CPI). Therefore, it can be concluded that, given either a specific or several separated population(s), the proposed algorithm has the capability to generate an optimized loci set that can be utilized in both identity testing and paternity testing with minimized number of loci. We also compared the study of Kemp et al. [16] on cattle, goat and sheep. Kemp identified 97 loci for individual identification across the three species. Through our algorithm optimized, 18 loci are satisfactory for this task. To summarize, our algorithm can be used for individual identification (on human) or across groups. After comparison with existing research, our results are better than previous studies.

## Data Availability

The source and on-line tools can be found in https://spe.deepomics.org

## Abbreviations

CPD, $\mathbb {C}(\mathbb {L})$: Combined power of discrimination
CPI: Combined paternity index
HE: Heterozygosity
MPF, $\mathbb {F}_{\ell }$: Maximum profile frequency
NGS: Next-generation sequencing
PD: Power of discrimination
PE: Power of exclusion
PI: Paternity index
PM, *M*_*ℓ*_: Probability of matching
RMP, $\mathbb {R}(\mathbb {L})$: Random-match probability
STR: Short tandem repeat
WGS: Whole genome sequence
